# Using Landscape and Bioclimatic Features to Predict the Distribution of Lions, Leopards and Spotted Hyaenas in Tanzania's Ruaha Landscape

**DOI:** 10.1371/journal.pone.0096261

**Published:** 2014-05-05

**Authors:** Leandro Abade, David W. Macdonald, Amy J. Dickman

**Affiliations:** 1 Department of Zoology, The Recanati-Kaplan Centre, Wildlife Conservation Research Unit, University of Oxford, Oxford, Oxfordshire, United Kingdom; 2 Ruaha Carnivore Project, Iringa, Tanzania; University of Lleida, Spain

## Abstract

Tanzania's Ruaha landscape is an international priority area for large carnivores, supporting over 10% of the world's lions and important populations of leopards and spotted hyaenas. However, lack of ecological data on large carnivore distribution and habitat use hinders the development of effective carnivore conservation strategies in this critical landscape. Therefore, the study aimed to (i) identify the most significant ecogeographical variables influencing the potential distribution of lions, leopards and spotted hyaenas across the Ruaha landscape; (ii) identify zones with highest suitability for harbouring those species; and (iii) use species distribution modelling algorithms (SDMs) to define important areas for conservation of large carnivores. Habitat suitability was calculated based on environmental features from georeferenced presence-only carnivore location data. Potential distribution of large carnivores appeared to be strongly influenced by water availability; highly suitable areas were situated close to rivers and experienced above average annual precipitation. Net primary productivity and tree cover also exerted some influence on habitat suitability. All three species showed relatively narrow niche breadth and low tolerance to changes in habitat characteristics. From 21,050 km^2^ assessed, 8.1% (1,702 km^2^) emerged as highly suitable for all three large carnivores collectively. Of that area, 95.4% (1,624 km^2^) was located within 30 km of the Park-village border, raising concerns about human-carnivore conflict. This was of particular concern for spotted hyaenas, as they were located significantly closer to the Park boundary than lions and leopards. This study provides the first map of potential carnivore distribution across the globally important Ruaha landscape, and demonstrates that SDMs can be effective for understanding large carnivore habitat requirements in poorly sampled areas. This approach could have relevance for many other important wildlife areas that only have limited, haphazard presence-only data, but which urgently require strategic conservation planning.

## Introduction

Apex predators such as lions (*Panthera leo*), leopards (*Panthera pardus*) and spotted hyaenas (*Crocuta crocuta*) play an important role in the regulation of ecological interactions and ecosystem health, substantially influencing lower trophic levels [1], [2]. As keystone species [3]–[5], they affect the density of mesopredators and natural prey [5], [6], and influence plant communities [7] by suppressing the effects of large ungulates on vegetation [8]–[10]. Their removal from the ecosystem can unleash trophic cascades [11], resulting in alteration of top-down regulations of the ecosystem [8], [12] and loss of biodiversity and species richness [12], [13]. Despite their ecological importance, large carnivores have experienced dramatic reductions in both population size and geographic range over the past century, necessitating urgent conservation planning for these species [14].

In Africa, high levels of human-induced carnivore mortality have been shown to be one of the most important factors leading to local extinction of large carnivores [15]–[17]. Areas adjacent to protected areas often experience particularly high human-carnivore conflict (HCC) and carnivore killing, acting as significant population sinks [18]–[20]. It is therefore vital to develop effective large carnivore conservation strategies both within and beyond the boundaries of protected areas. However, developing such strategies across an entire landscape requires an understanding of the eco-geographical preferences and therefore of the distribution of the target taxon [21]. Large carnivore distribution and habitat selection is largely determined by prey availability [22], [23], which in turn is affected by factors such as vegetation cover, water availability and elevation [23]–[25]. However, in many high-priority wildlife areas, researchers lack the time and resources needed to collect systematic presence-absence data on prey and carnivore distributions at the landscape level. They often rely upon opportunistic detections of species occurrence, and this presence-only data might not be well-suited to commonly-used techniques such as occupancy modelling [26], [27].

This issue is vividly illustrated by Tanzania's Ruaha landscape, which is a priority area for African carnivore conservation [28]. This landscape supports over a tenth of the world's lions [29], one of only four cheetah (*Acinonyx jubatus*) populations in East Africa numbering 200 adults or more [30], the world's third largest population of the endangered African wild dog (*Lycaon pictus*) [30] and globally important populations of leopards and spotted hyaenas [30], [31]. However, despite this global significance, the Ruaha landscape has received very little research attention and there are no published studies on large carnivore distribution and spatial ecology in this area, preventing the development of informed carnivore conservation plans. Such planning is particularly urgently needed given the extremely high level of human-carnivore conflict around Ruaha, which is strongly influenced by lion, leopard and spotted hyaena depredation upon livestock [32]. As in many important wildlife areas, researchers in Ruaha opportunistically gathered presence-only data on carnivore locations from camera-trapping and sightings across the landscape, and there is an urgent need to use this information as fully as possible, in order to predict wider patterns of carnivore presence and the potential for conflict with humans.

Recent species distribution modelling algorithms (SDMs) such as Maxent [33], Ecological Niche Factor Analysis (ENFA) [34] and Support Vector Machines (SVMs) [35], are potentially useful for determining species habitat suitability and distribution patterns from presence-only data, as they are able to deal with limited sample size and biased sampling (detailed explanation provided in the next section) [33], [34], [36], which is a common problem in studies involving large carnivores. SDMs incorporate an array of eco-geographic factors to predict species distribution based on habitat suitability [37], [38]. The models generate data which can be converted to maps of potential species distribution, which enable researchers to identify areas of particular importance for conservation and help inform future conservation planning strategies [38], [39]. Furthermore, identification of areas where people and carnivores are likely to overlap (e.g. if highly suitable habitat is located close to a park boundary) will help to identify potential hotspots of human-carnivore conflict. These data can be used to help target pre-emptive conflict mitigation strategies in high-risk areas, thus reducing the impact of conflict and retaliatory killing of large carnivores.

Therefore, this study aims to evaluate the potential distribution of three key large carnivores - lion, leopard and spotted hyaena - across the Ruaha landscape, based upon key environmental and bioclimatic features likely to influence carnivore habitat suitability in this ecosystem. Due to low sample sizes for African wild dogs and cheetahs, these species were not considered in the study. An ensemble modelling technique, derived from Maxent, ENFA and SVMs, was used to (i) identify the most significant environmental and bioclimatic factors influencing the distribution of each species and the overall assemblage of large carnivores; (ii) estimate the portion of the study site with highest suitability for harbouring large carnivores; and (iii) map out areas of conservation importance for these species. The ensemble modelling approach has been described as a reliable strategy for increasing the reliability of predictions for species distribution, since the final output incorporates areas of consistent prediction from all models [40], [41]. This study is the first to investigate the key factors affecting large carnivore distribution in the poorly known Ruaha region and to generate maps of likely presence, which also highlight risky zones for human-carnivore conflict. Our ensemble modelling approach can be employed in other priority wildlife areas for which opportunistically-collected presence-only data is available and which require urgent conservation planning.

## Methods

### Study Site

**Figure 1:**
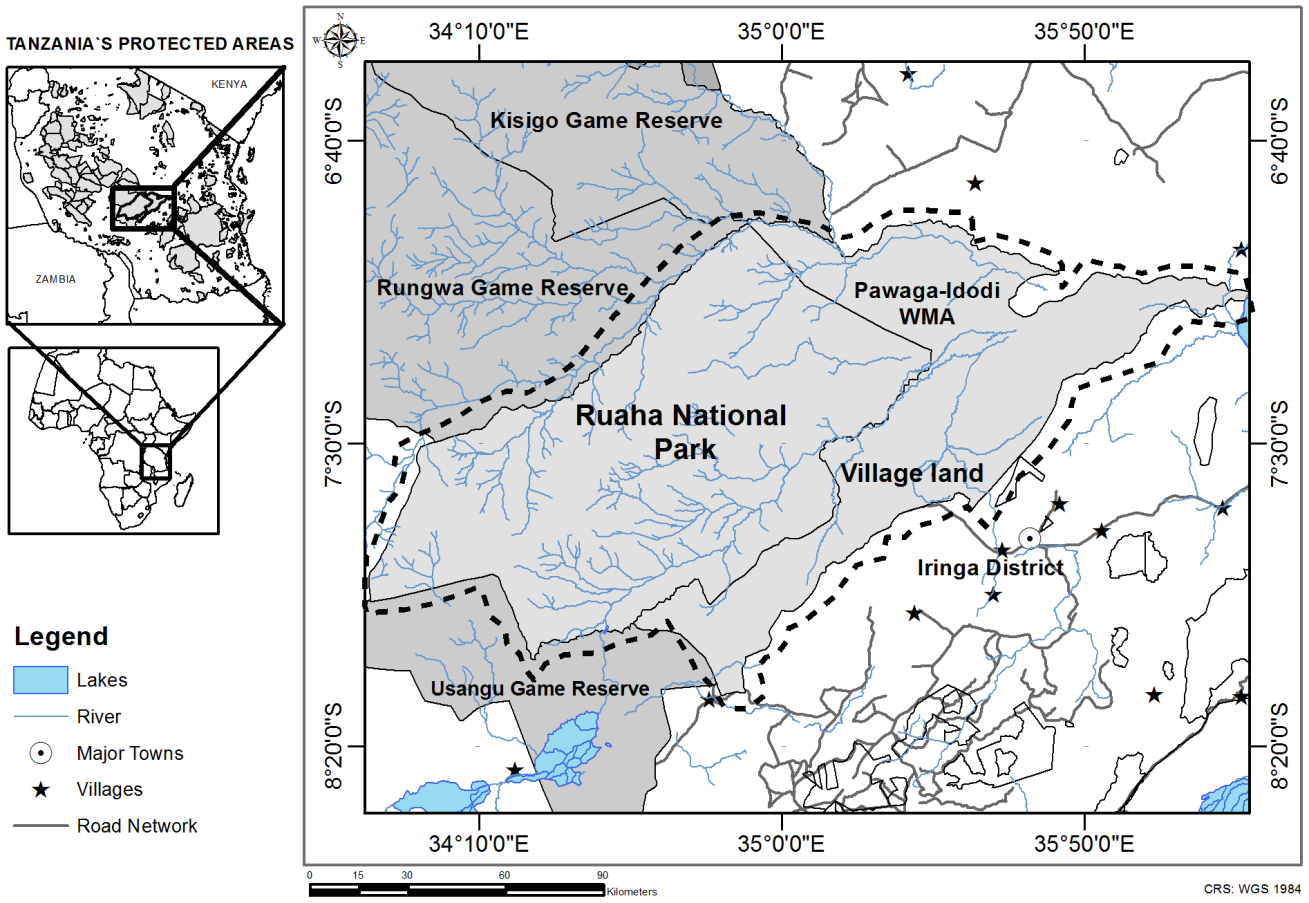
Map of the Rungwa-Ruaha region, highlighting the location of Tanzania's Ruaha landscape. Location of the Rungwa-Ruaha region, southern Tanzania, East Africa, composed of the Ruaha National Park, Pawaga-Idodi Wildlife Management Area, adjacent game reserves and village land. The dashed black line highlights the study area, the Ruaha landscape, comprised by the Ruaha National Park, Pawaga-Idodi Wildlife Management Area and village land.

The study area, hereafter named the Ruaha landscape, is located in the Rungwa-Ruaha region of Tanzania, and includes the Pawaga-Idodi Wildlife Management Area (PIWMA), Ruaha National Park (RNP) and adjacent village lands, between 6°40′00" to 8°20′00" S and 33°20′00" to 35°50′00" E (Figure 1), spanning across 21,050 km^2^. The whole Rungwa-Ruaha ecosystem encompasses more than 45,000 km^2^, and is considered to be one of the most biologically valuable ecoregions in the world due to its plant and animal richness [42].

The Rungwa-Ruaha region bears an outstanding guild of large carnivores, harbouring an estimated population of 3,779 lions, representing one of four lion strongholds in East Africa [29], a significant population of leopards and spotted hyaenas, the third largest population of the endangered African wild dog in the world [30], and one of the only four Eastern African cheetah populations supporting at least 200 adults [30]. Due to its importance for threatened large carnivores, this area has been considered a priority for African carnivore conservation [28]. At the heart of the Rungwa-Ruaha region is the Ruaha National Park (RNP), which at 20,226 km^2^ is Tanzania's largest Park [43]. RNP was created in 1964, expanded in 2009, and is listed as category II by the IUCN [44]. Large parks such as Ruaha are designed to protect a functioning ecosystem and the large-scale ecological processes that would disappear from small protected areas. These protected areas also represent important biodiversity corridors, connecting and protecting wide-ranging and migratory species which could not be conserved in smaller and more isolated areas [44].

The climate of the region is semi-arid to arid, with rainfall peaks occurring between December to January and March to April, and an average annual rainfall of 500 mm [45]. The temperature ranges from 15 to 35°C [46]. The altitude ranges from 696 to 2,171 m [47]. The vegetation cover is a mosaic of typical East African semi-arid savannah and northerly Zambesian *miombo* woodland [48], *Acacia* sp, *Combretum* sp. and *Commiphora* sp. Land-use varies from woodland to grassland and cultivated landscapes, with at least 17 different types of vegetation classes [47]. The Ruaha River is the main water supply in the study area, providing key resources for wildlife, attracting species towards the park borders with the PIWMA and village land. During the driest periods of the year, the river becomes the most important water source for wildlife and livestock.

### Species distribution data

Georeferenced presence-only points of carnivore locations were collected from 2010 to 2013 from RNP, village lands and the adjacent PIWMA using diverse techniques such as opportunistic direct observation, scat identification and camera-trapping. The camera-trapping data were derived from surveys carried on in the study site during 2011 and 2013. During the surveys, single station Reconyx cameras were placed along animal trails, and each camera was installed at least 1 km apart from each other, surveying different habitat types within the study site. To avoid issues of pseudoreplication within species sampling [49] and minimize spatial correlation, a Global Moran's I test was performed to assess the distribution pattern of the collected presence points across the study area [50]. For leopard and spotted hyaena, no spatial buffering of point selection was necessary due to low spatial autocorrelation among points. For lions, only single presence points located at least 3 km apart were considered, which decreased spatial autocorrelation. The data collection inside Ruaha National Park, village land and PIWMA was conducted in strict accordance with the research permit issued by the Tanzania Wildlife Research Institute (TAWIRI) and the Commission for Science and Technology (COSTECH).

### Ecogeographical variables

The ecogeographical variables (EGVs) were selected according to their potential influence on the distribution of each species [51]–[55], and were divided among landscape, bioclimatic and human disturbance features (Table 1). Prior to running the models, the set of EGVs was first submitted to a Pearson correlation test using the Correlation function available on ENMTools software v. 1.3 [56], to avoid issues of multicollinearity among variables. Guisan et al. [57] showed that highly correlated covariates could be considered as non-significant by the model, even significantly contributing for the model output when considered individually. Therefore, the selected predictors were those minimally correlated (<±0.85 [58]). Elevation data were extracted from the Shuttle Radar Topographic Mission [59], and slope was derived from elevation, using the function Slope in ArcGIS v. 10.1. The mean rainfall index (mm) for the study site was downloaded from the WorldClim database v. 1.04 [60]. The ‘distance to water bodies (km)’ and ‘distance to human households (km)’ rasters were generated using the Euclidean Distance function in ArcGIS v. 10.1 based on the presence of water bodies in the study site [61], and the location of households mapped by WildCRU's Ruaha Carnivore Project (RCP) in the study site. Due to scarcity of data on the local distribution of wild prey such as ungulates and other herbivores, a Normalized Difference Vegetation Index (NDVI) layer was considered. NDVI is related to photosynthesis and vegetation productivity [62], [63], and has been incorporated into models [55] as a proxy to identify potential landscape patches with increased biomass of ungulates and other herbivores [64], [65].

**Table 1:** Set of ecogeographical variables (EGVs) used for modelling the potential distribution of lions, leopards and spotted hyaenas in Tanzania's Ruaha landscape.

Ecogeographical Features	Source	Original Resolution
*Bioclimatic variables*		
Annual precipitation	WorldClim database [60], v. 1.04. http://www.worldclim.org/current	30 arc-seconds (∼1 km)
*Landscape features*		
Digital elevation model	Shuttle Radar Topography Mission. http://www.landcover.org/data/srtm/	3 arc-seconds (90 m)*
Slope	Derived from digital elevation model	-
Normalized Difference Vegetation Index – NDVI	MODIS Terra - MOD13A. http://glovis.usgs.gov/	5001 m*
Vegetation Continuous Fields - VCF	ftp://ftp.glcf.umd.edu/modis/VCF/Collection_5/	250 m*
Geology - Cation Exchange Capacity	http://www.isric.org/data/soil-property-maps-africa-1-km	1 km
Distance to water bodies	Derived from http://www.fao.org/geonetwork/srv/en/metadata.show?id=2002&currTab=simple	-
*Human Disturbance*		
Distance to human households	Derived from Ruaha Carnivore Project Data	-

*Raster files converted to ∼1 km×1 km cell size.

The NDVI raster incorporated into the models was the mean value calculated for the study site (using the function Raster calculator in ArcGIS v. 10.1), derived from compilation and processing of NDVI rasters from Terra (EOS AM) satellite images generated between March 2011 and December 2012. Information on vegetation cover (vegetation continuous fields - VCF) was also considered. VCF depicts the landscape surface as gradations of three components of ground cover, (1) percent tree cover, (2) percent of non-tree vegetation, and (3) bare soil [66]. A raster containing information on the cation exchange capacity of the soil was incorporated as it is also related to vegetation cover and primary net productivity. Higher cation exchange capacity is related to increased soil capacity to retain nutrients, which contributes to soil fertility and plant productivity [67], and positively influences large ungulate biomass and lion distribution [68], [69].

**Figure 2:**
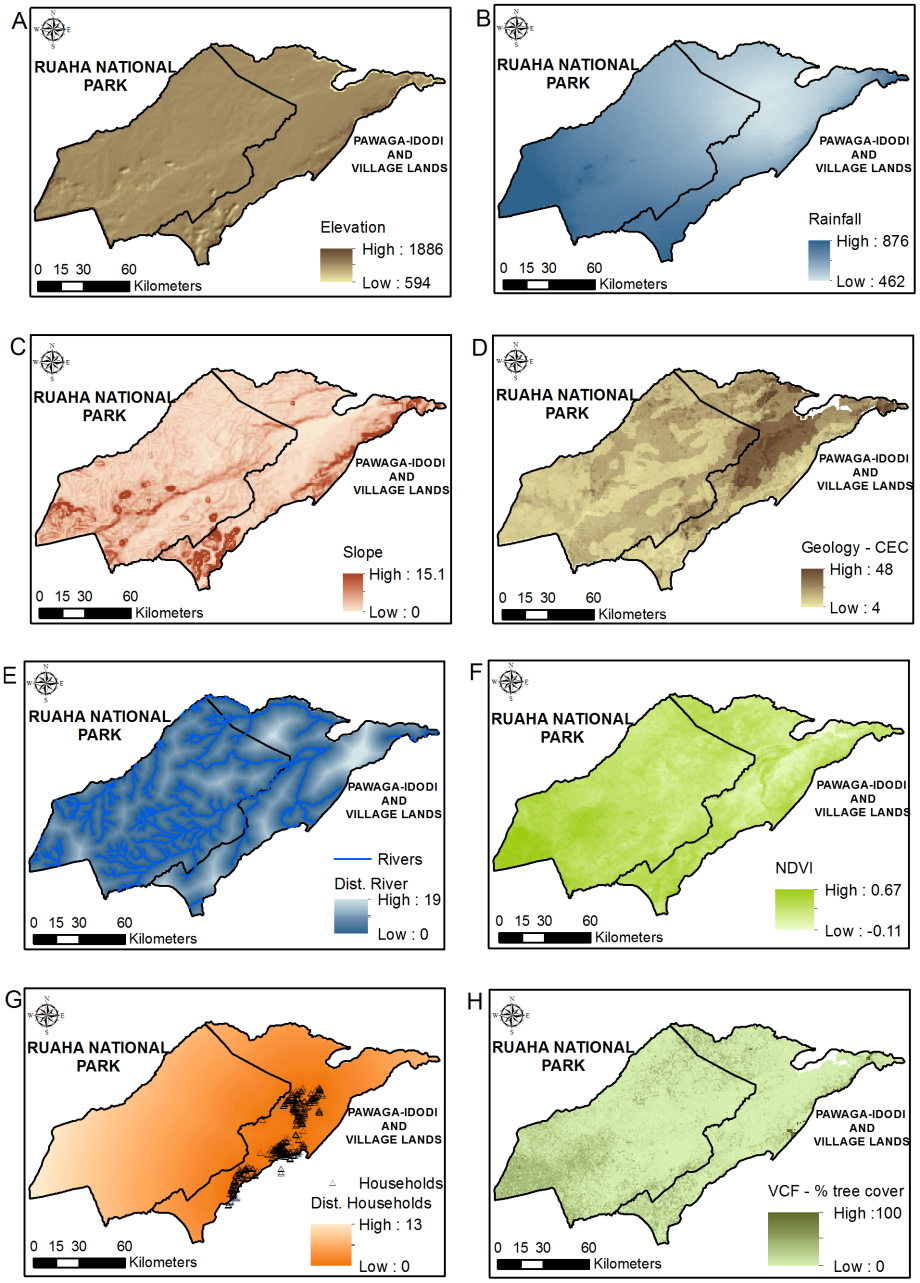
Representation of variables used for predicting the distribution of large carnivores in the Ruaha landscape. Representation of raster files used for the predictive modelling of the distribution of lions, leopards and spotted hyaenas in the Ruaha landscape. A. Elevation (m), B. Rainfall (mm), C. Slope (degrees), D. Geology – cation exchange capacity, E. Distance to rivers (km), F. NDVI, G. Distance to households (km) and H. VCF (% tree cover).

The raster files were converted in habitat-grid cells of approximately 1 km×1 km resolution according to their original resolution size, and reprojected to UTM 36 S (Table 1, Figure 2). Spatial data preparation and raster analyses were conducted in ArcGIS v.10.1 [70] and IDRISI Selva [71], and statistical analyses in R v. 2.15.1 [72]. ENFA modelling was conducted in Biomapper v. 4.0.7.373, Maxent in Maxent v. 3.3.3e and SVM in openModeller Desktop v. 1.1.0.

### Building the predictive models

Prior to spatial modelling, the presence-only location points related to each species were randomly divided into training (70%) and testing (30%) data to allow post-hoc validation of the resulting models. ENFA, SVMs and Maxent algorithms were used to build the final ensemble model [40], [41] to determine the potential distribution of lions, leopards and spotted hyaenas across the Ruaha landscape. A nested model incorporating all large carnivore species together was also considered in order to compare the potential influence of the EGVs on the distribution of the overall assemblage of large carnivores. Although many different algorithms are available, these predictive algorithms were selected based, especially, on their high predictive power, and due to their capabilities of performing well relying solely on presence-only data [33], [34], [73]. This is relevant as, often, distribution models have to rely on data from species surveys, which cannot identify areas of total absence of occurrence for a particular species within its distribution range, especially for large carnivores [74]. Therefore, the capacity to use presence-only points obviates the risk that models incorporate unreliable absence records (which could lead to unrealistic and misleading scenarios of species probability of occupancy and distribution [74], [75]).

The ENFA algorithm is based on Hutchinson's ecological niche concept [34], [76], which is described as a multidimensional space in a hyper-volume comprising ecological variables that allows an organism to survive and reproduce [34], [76], [77]. ENFA calculations are similar to a principal component analysis, and summarize the species preference for habitat types in two distinct factors, marginality and specialisation, which measure the habitat used from the overall habitat available [78]. The first factor represents species global Marginality (M), comparing the deviation of all the environmental conditions where the species occurs (species distribution) to those found in the study area (global distribution) [78], and ranges from 0 to 1, with higher values indicating that the species inhabits a very particular habitat type in relation to the reference habitat [34]. ENFA calculates a ‘marginality coefficient’ relating the degree of correlation between each variable to the global marginality factor, identifying species preferences for particular EGVs. High absolute marginality value of a coefficient indicates that the species favours that particular EGV more than the mean value available in the habitat, and the more this particular variable contributes to the global marginality. A negative marginality coefficient indicates that the species favours lower-than-mean values of a particular EGV than those found in the habitat [79]. The second factor represents species global Specialisation (S), which is a measure of the ratio of the variance in global distribution to that observed in the species distribution [34] (i.e. species' niche restrictiveness [80]), with values above 1 indicating certain levels of specialisation by the species [34]. Moreover, ENFA provides an overall index of species tolerance (global Tolerance, 1/S) which varies from 0 to 1, with values close to 1 indicating that the species tolerates large variations from its optimum conditions [81], is widely distributed in the study site [79], and show broader niche breadth [82]. To facilitate interpretation of the results generated by ENFA, the mid-point of the Global Marginality and Global Tolerance indices (i.e. 0.5) was used as a threshold, with values above the threshold for (M) indicating species preference for particular habitat types, and those for (1/S) indicating low niche restrictiveness and broader niche breadth. No threshold was defined for (S) as the values vary from 1 to infinity. The influence of each EGV was also based on a threshold choice, with the absolute value of the marginality coefficients above the mid-point (i.e. >0.5) indicating stronger preference for a particular EGV.

Support vector machines (SVMs) algorithms have recently become adopted in the field of spatial modelling of species distribution in order to assess niche suitability [36], [83]–[85]. SVM is a powerful tool to deal with data uncertainty, sampling autocorrelation and presence-only datasets [36], dealing well with problems commonly related to ecological studies such as small sample size and incidentally-collected (haphazard) data. The algorithm is part of a non-probabilistic pattern recognition classifier [85]. It relies on a kernel-based function [35] to classify objects in a multidimensional optimal hyperplane, i.e. one with maximized margin of separation between two data clusters [86], [87]. Although a robust predictive method, it can be challenging if used in isolation as it does not tabulate the potential contribution of each variable as Maxent and ENFA do, thereby limiting identification of key features associated with greater habitat suitability [83].

Maxent is a machine learning method used to estimate probability of distribution, based on the principle of maximum entropy [33]. Maxent will predict the probability of species distribution under the most dispersed scenario assuming all the environment constraints affecting species presence were taken into consideration during the calculations [33], [73]. The Maxent models were run using the settings defined by Phillips *et al*. [33]. As in the ENFA algorithm, a threshold above the mid-point of variable contribution (i.e. >50% contribution) was chosen to determine those variables strongly influencing species habitat suitability and therefore, probability of occurrence.

### Model validation

The performance of each independent model was evaluated by calculating the area under the receiver operating characteristic (ROC) curve, abbreviated to Area Under the Curve (AUC) [75], [88] in IDRISI Selva. The ROC curve is calculated based on values of sensitivity (correct discrimination of true positive location points) and specificity (correct discrimination of true negative absence points) of the model. The AUC ranges from 0.5 (random) to 1.0 (perfect discrimination), providing reliable estimation of model fitness as it compares the likelihood of occurrence with the true presence data used as reference [89], [90]. Models showing AUC values >0.7 are considered fair and those with scores >0.9 are considered highly accurate [91], [92].

Each modelling approach has limitations and potential errors inherent to their algorithms and may show different levels of efficacy and performance while predicting species distribution [40], [41], [93]. Developing consensus models is as a good strategy for overcoming model uncertainties and increasing the reliability of predictions, since the final output incorporates areas of consistent prediction from all models [41], [83]. Here, a consensus model (ensemble model) was calculated using the weighted average of the resulting internal AUC values of each model (training AUC), as described in Marmion *et al*. [40], and used by Rodríguez-Soto *et al*. [21] to predict the distribution of jaguars in Mexico. Model performance was also assessed using the external AUC (testing AUC), resulting from the models developed using the independent set of presence-only points (testing data), as described in Zarco-Gonzales [83]. The consensus model was also assessed according to the resulting weighted AUC value.

Finally, the ensemble model outputs were converted into maps of habitat suitability for species occurrence. These maps depict a gradient of suitability across the landscape, in which each grid cell of the map has an associated value of habitat suitability, and therefore probability of species occurrence, varying from 0 to 100, with highly suitable grids cells closer to 100. To allow identification of the most important areas for species occurrence, and following [21], [83], [94], highly suitable grid cells were defined as those with values above the species median suitability, considering the median value of the location points used to train the models. According to Liu *et. al* [94], the median is a meaningful threshold choice as it does not assume a symmetric distribution (i.e. normal distribution) for habitat suitability across the landscape. Nevertheless, a lower threshold value (>50% probability of occurrence) was also considered whilst building the predictive map in order to identify areas with lower suitability but potentially used by each species. This enabled comparisons of distinct model outputs based on different threshold values, with implications for conservation strategies. Highly suitable grid cells were mapped in order to identify potential core areas for species occurrence and conservation importance. A linear model was used to assess the distribution patterns of highly suitable cells according to each carnivore species. In addition, the distance of these grid cells to village lands was calculated, highlighting zones of potential overlap between large carnivores and human activities which are likely to be human-carnivore conflict hotspots.

## Results

### Species distribution data

In total, 122 presence points were considered for leopards (118 points from direct observations; 2 from scat identification; 2 from camera-trapping), 93 for spotted hyaenas (88 from direct observation; 5 from camera-trapping) and 59 for lions (52 from direct observation; 7 from camera-trapping). The majority of carnivore location points considered for the models were collected within RNP, with a total of 96% (n = 117) of the leopard points collected in RNP, 97.9% (n = 91) of the locations for spotted hyaenas, and 88.1% (n = 52) for lions, with the remainder collected outside the park (i.e. in the village lands and PIWMA). Although few location points were collected outside the National Park, they helped the model to incorporate landscape heterogeneity, especially regarding information on carnivore occurrence in relation to close proximity to human households.

### Model performances

Overall, the SDMs performed well in terms of predicting the distribution of all large carnivores across the study area, with all models showing AUC values above 0.7 (Table 2). Maxent outperformed ENFA and SVMs in predicting large carnivore distribution, both for individual species and when the three were nested together (Table 2). ENFA was the lowest-performing algorithm for both individual carnivores and the nested model, while the ensemble model performed well, with AUC values only slightly lower than from Maxent alone.

**Table 2:** Performance of algorithms used to predict the distribution of leopards, lions and spotted hyaenas across Tanzania's Ruaha landscape.

Algorithm	Species	Training AUC	Testing AUC
*ENFA*	Nested	0.753	0.765
	Leopards	0.756	0.822
	Lions	0.750	0.622
	Spotted Hyaenas	0.822	0.848
*Support Vector Machines*	Nested	0.892	0.883
	Leopards	0.905	0.902
	Lions	0.863	0.701
	Spotted Hyaenas	0.886	0.904
*Maxent*	Nested	0.947	0.921
	Leopards	0.949	0.957
	Lions	0.873	0.753
	Spotted Hyaenas	0.944	0.960
*Ensemble*	Nested	0.907	0.900
	Leopards	0.921	0.934
	Lions	0.852	0.706
	Spotted Hyaenas	0.921	0.942

Significant models showing AUC>0.7.

### Nested model

**Figure 3:**
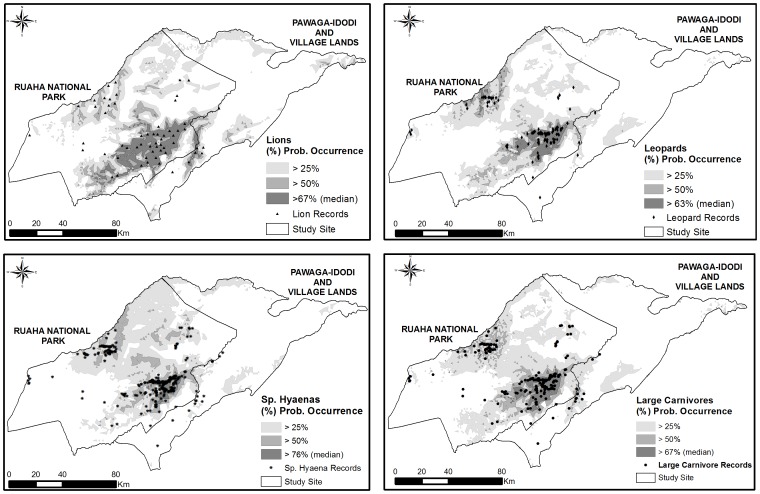
Predictive map of the potential distribution of large carnivores in Tanzania's Ruaha Landscape. Map of potential distribution of large carnivores across Tanzania's Ruaha landscape. The maps were generated using ensemble modelling approach based on the outputs of Maxent, ENFA and SVMs. The colour gradient indicates probability of species occurrence, with darker areas representing the highly suitable areas (h.s.> species median suitability) for species occurrence.

The ENFA analysis suggested that, if assessed as an assemblage (i.e. no discrimination among species), the large carnivores had a global Marginality value (M = 0.446) slightly below the pre-defined threshold of 0.5, suggesting that they did not select for an overly narrow set of ecogeographical traits. The global Tolerance value (1/S = 0.414; Table 3) suggested they showed low relative ecological flexibility to variations in the optimal environmental conditions available at the study area. The ENFA algorithm suggested that habitat suitability for the assemblage of large carnivores increased mostly with proximity to water bodies (Table 4). NDVI, VCF and altitude showed some contribution to species marginality and habitat suitability, but did not strongly influence large carnivore distribution (Table 4). Maxent modelling identified annual precipitation as the most important variable influencing large carnivore habitat suitability, followed by increased distance to human settlements and proximity to rivers. However, none of the predictors seemed to exert a strong influence on species distribution (Table 4). From the overall potential distribution of large carnivores across the Ruaha landscape, a total of 2.18% (442 km^2^; median suitability >67% habitat suitability) of the study area was estimated as highly suitable for at least one of the large carnivores according to the ensemble model (Table 5). From this total, 0.18% (0.89 km^2^) of the predicted highly suitable grid cells for large carnivores were located within village land. Highly suitable areas were mainly identified in the mid-eastern portions of the National Park, close to the borders with village land (Figure 3). Using a lower threshold value (>50% habitat suitability), a total of 6.6% (1, 388.9 km^2^) of the study area was mapped as suitable for the species, with suitable areas scattered in the mid-eastern and west portions of the National Park. A total of 3.41% (47.43 km^2^) of these suitable grid cells for large carnivores were located within village land (Figure 3).

**Table 3:** Indices for species niche global marginality and tolerance according to Ecological Niche Factor Analysis (ENFA).

Species	Marginality (M)	Specialisation (S)	Tolerance (1/S)
*Nested*	0.446	2.416	0.414
*Leopards*	0.492	3.045	0.328
*Lions*	0.436	2.463	0.406
*Spotted Hyaenas*	0.578	3.471	0.288

**Table 4:** Contribution of ecogeographical variables to large carnivore distribution in Tanzania's Ruaha landscape according to ENFA and Maxent.

Species	Variables	ENFA		Maxent
		Marginality Coefficient	Specialisation Coefficient	(%) Contribution
*Nested*	Dist. River	−0.68	−0.25	13.1
	NDVI	−0.36	−0.20	5.8
	VCF	−0.35	0.63	4.2
	Altitude	−0.35	0.42	12.1
	Slope	−0.27	0.01	1.4
	Geology	−0.22	−0.12	3.6
	Annual precipitation	0.17	0.40	44
	Dist. Settlements	−0.09	−0.39	15.8
*Leopards*	Dist. River	−0.62	0.28	12.7
	NDVI	−0.47	0.12	2.4
	VCF	−0.37	−0.38	2.1
	Altitude	−0.36	−0.58	13.8
	Slope	−0.25	−0.01	1.2
	Geology	−0.20	0.15	4.5
	Annual precipitation	0.14	−0.39	49.7
	Dist. Settlements	−0.08	−0.50	13.7
*Lions*	Dist. River	−0.74	0.19	30.1
	NDVI	−0.22	0.01	1.1
	VCF	−0.32	0.84	6.8
	Altitude	−0.36	−0.27	17.2
	Slope	−0.27	−0.32	3
	Geology	−0.12	0.15	2.9
	Annual precipitation	0.29	−0.06	30.4
	Dist. Settlements	−0.10	0.21	8.5
*Spotted hyaenas*	Dist. River	−0.59	−0.21	14.6
	NDVI	−0.49	−0.07	1.6
	VCF	−0.33	0.24	21.5
	Altitude	−0.35	0.57	9.8
	Slope	−0.30	0.08	2.7
	Geology	−0.26	−0.16	0.6
	Annual precipitation	0.03	0.38	32.2
	Dist. Settlements	−0.16	−0.62	17.1

Estimated marginality and specialisation coefficients according to ecogeographical variables by Ecological Niche Factor Analysis (ENFA) and % variables contribution to species distribution according to Maxent. Negative signs indicate preference towards lower values of a particular EGV [34].

**Table 5:** Total estimated habitat suitability area/species according to the ensemble model output.

Species	Suitable Area km^2^ (% of Study Site) h.s.>25%	Suitable Area km^2^ (% of Study Site) h.s.>50%	Suitable Area km^2^ (% of Study Site) h.s.> median
*Nested*	5, 580.7 (26.5)	1, 388.9 (6.6)	442 (2.1)
*Leopards*	4, 904.1 (23.3.)	1, 260.9 (6.0)	510.1 (2.4)
*Lions*	6, 161.5 (29.2)	2, 214.9 (10.5)	1, 010.4 (4.8)
*Spotted hyaenas*	5, 745.4 (27.3)	1, 195.6 (5.7)	181.7 (0.8)

Highly suitable grid cells with increased probability of species occurrence were defined as those with values above the species median suitability and probability of occurrence (h.s.> median). Total area study site: 21,050 km^2^.

### Leopards

The ENFA results suggested that leopards showed certain selectivity for specific habitat types within the study area, but were slightly below the pre-defined marginality threshold and, overall, did not select a particularly narrow set of habitat conditions (M = 0.492). The species also showed limited tolerance for large variations in the optimal conditions of EGV available in the landscape (1/S = 0.328) (Table 3). Leopards showed higher marginality and lower tolerance than lions, though less specificity and more tolerance to habitat changes than spotted hyaenas. According to the algorithm, habitat suitability of leopards was associated with proximity to rivers (Table 4). Areas of relatively low primary net productivity, open canopy and lower altitudes also contributed to habitat suitability, but not as strongly as distance to water bodies (Table 4). The Maxent results suggested that leopard distribution in Ruaha was influenced by a positive index of annual precipitation, with that variable alone contributing almost 50% to the probability of species occurrence. Even though the distribution of leopards seemed to be spread widely across the landscape in terms of the overall habitat suitability for the species (Figure 3), the ensemble model estimated that only approximately 2.4% (510.1 km^2^) of the study site was highly suitable (> median suitability) for leopard occurrence (Table 5), with no suitable areas for the species occurring outside the National Park. The most suitable areas for leopards were those located in the mid-eastern portions of RNP. Using a lower threshold selection (>50% habitat suitability), 6% (1, 260.9 km^2^) of the study site was mapped as suitable for leopard occurrence (Table 5), and from this total, 0.7% (8.9 km^2^) was located within village land (Figure 3).

### Lions

The ENFA algorithm suggested that lions did not show a strong preference for particular habitat conditions (M = 0.436), although they had relatively low tolerance to changes in the environmental conditions composing the habitat (Table 3). The species showed the lowest marginality and highest tolerance among any other carnivore species assessed, suggesting that, in this particular landscape, lions do not select for very specific habitat types. ENFA also suggested that, in the Ruaha Landscape, habitat suitability for lions was strongly influenced by proximity to water sources, while lower elevation, and open canopy, also made a slight contribution to habitat suitability (Table 4). The Maxent algorithm related habitat suitability for lions to annual precipitation and proximity to water sources (Table 4). Based on the median suitability threshold approach, 4.8% of the study area (1,010.4 km^2^) was predicted to be highly suitable for lions (Table 5). From this total, 4.5% (45.6 km^2^) of the highly suitable areas for lions were mapped within village lands, overlapping with human-dominated areas. Using the mean value (>50%) as threshold for habitat suitability, 10.5% of the study site (2, 214.9 km^2^) was identified as suitable for the species (Table 5), with 11.4% (252.3 km^2^) of this total located within village land. The majority of areas with higher probability of lion occurrence (i.e. highly suitable) were located in the most central and eastern portions of RNP, close to the boundaries between RNP and village land. In addition, areas of increased habitat suitability were also identified close to the north-western borders of RNP (Figure 3).

### Spotted hyaenas

The ENFA algorithm suggested that probability of occurrence of spotted hyaena was related to certain habitat types (M = 0.578), and that the species showed the most limited tolerance to large deviations in environmental features, and narrow niche breadth (1/S = 0.288). They exhibited lower tolerance compared to any other carnivore examined, and the highest specificity for particular habitat types. In addition, the ENFA results suggested that spotted hyaena habitat suitability was strongly influenced by distance to rivers (Table 4). Areas of decreased net productivity, low altitude and vegetation cover also had higher habitat suitability for the species (Table 4). The Maxent modelling linked an increased probability of hyaena occurrence with higher annual precipitation, with little influence of vegetation cover, and distance to settlements (Table 4). The species showed the most limited distribution among all the large carnivores based on the amount of highly suitable cells estimated by the ensemble model, with highly suitable areas covering only 0.8% of the study area (181.7 km^2^, Table 5). This limited distribution is likely to reflect the elevated median of habitat suitability for species occurrence used in the analysis (h.s.>76% species median suitability and probability of occurrence). Highly-suitable areas for spotted hyaenas were those located in the eastern sections of RNP, with 97% of the total occurring within 30 km of the Park-village border. No areas with increased habitat suitability were identified outside the National Park. Using the mean value of habitat suitability (h.s.>50%) as a threshold, 5.7% (1, 195.6 km^2^) of the study site was mapped as suitable for spotted hyaenas (Table 5), with suitable areas mainly located in the east part of RNP, and a few patches scattered across the mid-western portions of the park. Under this threshold, 1% (12.5 km^2^) of the suitable areas mapped for spotted hyaenas was located within village land (Figure 3).

### Distribution of highly suitable patches

**Figure 4:**
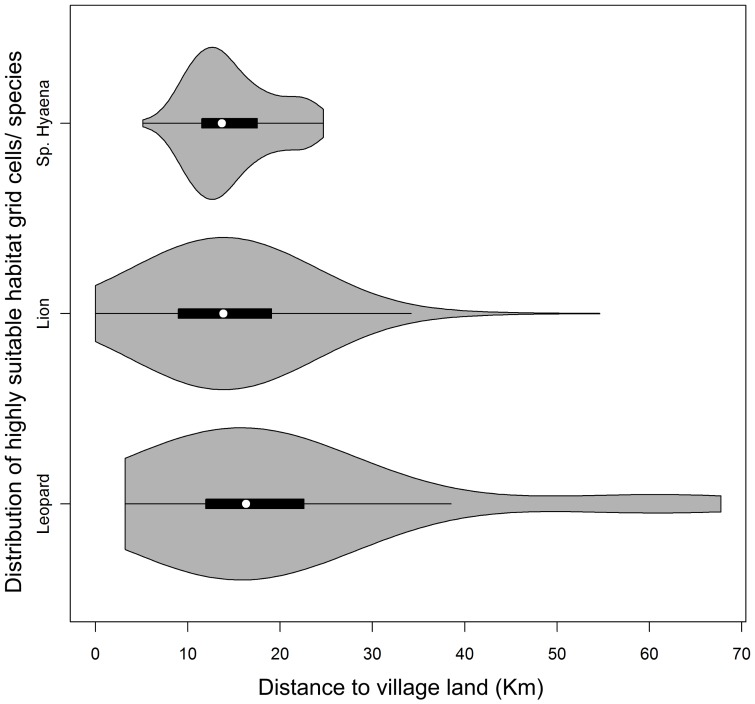
Distribution of highly-suitable areas for large carnivores in relation to proximity to village lands. Distribution of grid cells deemed highly suitable for large carnivores in relation to proximity to village land. The grey areas represent the probability density of the data. The horizontal black bar represents the first-to-third interquartile range, and the horizontal black line represents the 1.5 times the interquartile range. The median is represented by the white dot.

In total, 1,702 km^2^ (8.1% of the study area) emerged as highly suitable for all three carnivores collectively. Of this area, 95.4% (1,624 km^2^) was located within 30 km of the border between the Park and village land (Figure 4). According to the results of the linear model, the distribution of highly suitable cells varied significantly according to each large carnivore species, with areas of high suitability for spotted hyaenas occurring closer to the park boundaries than for lions and leopards (p<0.001) (Figure 4). The results also suggested that highly suitable habitats for leopards were patchier and more widely distributed across the study area than those for lions and spotted hyaenas (Figure 4). The proximity of highly suitable grid cells to village land is a cause for conservation as it suggests that large carnivores in Ruaha, especially spotted hyaenas and lions, are likely to occur in areas located close to human-dominated land. This increases both the risk of HCC and the likelihood that retaliatory killing will also impact carnivores within the Park.

## Discussion

Species distribution modelling proved effective at using opportunistically-collected data from Ruaha to provide the first data on carnivore habitat preferences and likely distributions across the wider landscape. This modelling showed that lions, leopards and spotted hyaenas all showed intermediate to high levels of specialisation, relatively narrow niche breadth and low ecological tolerance for large deviations from optimal environmental conditions available. Interestingly, in this landscape, the lion was the most tolerant species to changes in the environmental conditions, and showed greater niche breadth than the other carnivores, followed by leopard and spotted hyaena. The most common features influencing the probability of occurrence of large carnivores in the Ruaha Landscape were proximity to water bodies and positive index of annual precipitation for all the species assessed, corroborating previous studies which related habitat suitability of lions [23], [95], leopards [96] and spotted hyaenas [97] to areas of increased proximity to rivers and water bodies.

### Leopards

Increased habitat suitability for leopards has been related to proximity to water sources [98], [99], areas covered by thick bushes and forest types [1], [100], and with positive NDVI [55]. This study corroborates the importance of water availability in terms of habitat suitability for leopards, though in the Ruaha landscape the species avoided areas of increased net productivity, instead favouring habitat types with less vegetation cover. This pattern was also reported in Phinda Reserve, South Africa [101], with leopards favouring habitats of open-to-intermediate vegetation cover. These habitat types provide enough cover for hunting without interfering with prey detection, increasing hunting success [101]. Even though elevation has been suggested as a factor influencing habitat selection by leopards [1], enabling them to avoid competition with lions, our results suggest an overlap between all three large carnivores, as lions and spotted hyaenas also favoured lower altitudes. This pattern is potentially related to the increased distribution of wild prey around perennial water sources in lower elevation ranges. The influence of rainfall in habitat suitability supports previous studies which linked high precipitation to increased vegetation cover (i.e. grazing fields) and biomass of key leopard prey [101], [102], resulting in high hunting success, cub survival rates and reproductive success [96]. It is important to note that few sample points were collected in either the driest or hilly areas of the study site, which could limiting the models in identifying these areas as highly suitable for the species. It is therefore advisable that model validation should be conducted in the study area to assess whether these areas could potentially be suitable for the species (type I error).

### Lions

Lion potential distribution was largely influenced by proximity to rivers, which, as with leopards, is likely linked to the increased presence of water-dependent prey species in the surroundings of water bodies [103], [104], as reported in Hwange National Park in Zimbabwe [95] and Serengeti National Park in Tanzania [105]. Increased habitat quality for lions is known to be determined by proximity to water sources and seasonal rainfall [106], as these areas harbour higher availability of wild prey which increases lion hunting success, reproductive success and cub survival, characterizing these sites as population sources [106]. The results of this study support those of Davidson's *et al*. [107] which described surface water as a passive trap for prey, strongly influencing lion distribution. The influence of positive rainfall on habitat suitability is unsurprising as precipitation increases net primary productivity and water availability, affecting the distribution and availability of ungulates [108] and therefore lions [107], [109].

### Spotted hyaenas

Hyaenas are commonly portrayed as highly adaptable, showing relatively high plasticity to habitat disturbances [110], [111]. However, our findings suggest that hyaenas selected for particular habitat types, showed intermediate levels of ecological flexibility, and, even though the species does not require extreme niche conditions, it had relatively lower tolerance for large deviations from its optimal environmental conditions than lions and leopards.

As for lions and leopards, the preference of spotted hyaenas for areas close to water is probably due to higher prey availability and preferential denning sites in those locations [97]. However, on this note, it is important to highlight that, in the study site, due to issues of accessibility during surveys, sampling tended to rely on main roads which were those closer to water bodies, which could bias the observations of large carnivores towards rivers and areas of easy accessibility. Therefore, even though other studies corroborate our findings concerning the distribution of the carnivores studied [96], [107], [112], further sampling in remote and more arid areas of the study site would provide a better understanding of large carnivore spatial distribution and the influence of these variables in habitat suitability for the species assessed. The avoidance of highly productive areas found in the present study was also observed in spotted hyaenas from Kenya's Maasai Mara Game Reserve, where they preferred shrublands and areas of intermediate vegetation cover over forest [97]. Rainfall can significantly affect habitat suitability and population trends, as it influences hyaenas' feeding behaviour, demography, recruitment and intensity of conflict with humans. Cooper *et al*. [113] observed that oscillation in rainfall affected prey availability and led to immediate changes in hyaenas feeding behaviour, especially due to interference in the dynamics of ungulate migration. High precipitation has also been related to low recruitment, due to increased juvenile mortality and high human-carnivore conflict induced by fluctuations in prey availability [16].

### Distribution of highly suitable patches

The small portion of the study site classed as highly suitable habitat for large carnivores is probably due to the high median values used as thresholds for selection of highly suitable habitats. Even though they generate a more conservative distribution, threshold decisions based on predicted probability/suitability, such as the median suitability adopted in this study, can provide more reliable cut-off point to determine habitat suitability than arbitrary selection of 50% probability of occurrence [21], [94], [114], as the latter assumes a normal distribution of habitat suitability scores [94]. However, according to the results presented in this study, the selection of lower threshold values enabled identification of potential areas for species occurrence in areas never surveyed beyond the boundaries of the National Park, and also within village lands, in close proximity to human habitations, depicting potential hotspots of HCC. This information is valuable, as it enables selection and prioritization of those areas with increased livestock risk to predation, where implementation of HCC mitigation strategies is most needed to lessen livestock depredation, and, ultimately, to reduce retaliatory carnivore killing. Therefore, we suggest that for further studies, the threshold choice should reflect the conservation purposes of the study, as higher values may generate over-conservative and inadequate maps for identification of the most important areas for large carnivore conservation.

### Influence of human disturbance on species distribution

The low influence of human density on carnivore habitat suitability in this study must be regarded with caution since it might reflect sampling bias in data collection. The majority (95.6%) of carnivore locations used for modelling were collected within the National Park, with few collected in areas of high human density. This lack of representation of carnivore presence points from village lands could prevent the model from accurately assessing the influence of human disturbance on habitat suitability for each species. Therefore, further sampling of carnivore presence in village lands would produce a better assessment of the influence of human disturbance on large carnivore habitat suitability in this area.

## Conclusions

According to this study, the habitat suitability and distribution of leopards, lions and spotted hyaenas in the Ruaha landscape was strongly influenced by proximity to rivers and relatively high annual precipitation. The areas of highest suitability for large carnivore occurrence were those located in the eastern sections of Ruaha National Park, within 30 km of the Park-village border, raising concerns about HCC. This study shows that ensemble modelling based on presence-only data can be a valuable tool in areas which lack systematic data on carnivores, but where maps of likely carnivore distribution and habitat use would help inform much-needed management and conservation strategies.
